# Hospital admissions in relation to body mass index in UK women: a prospective cohort study

**DOI:** 10.1186/1741-7015-12-45

**Published:** 2014-03-15

**Authors:** Gillian K Reeves, Angela Balkwill, Benjamin J Cairns, Jane Green, Valerie Beral

**Affiliations:** 1 Cancer Epidemiology Unit, University of Oxford, Richard Doll Building, Roosevelt Drive, Oxford OX3 7LF, UK

**Keywords:** Body mass index, Obesity, Hospital admissions, Health services, Attributable risk, Cohort study

## Abstract

**Background:**

Adiposity is associated with many adverse health outcomes but little direct evidence exists about its impact on the use of health care services. We aim to describe the relationship between body mass index (BMI) and rates of hospital admission in middle-aged UK women.

**Methods:**

Among 1,251,619 Million Women Study participants, 50- to 64-years old at entry into the study, routine data on hospital admissions were used to estimate hospitalization rates according to BMI after standardization for age, region of recruitment, socioeconomic status, reproductive history, smoking status, hormonal therapy use and alcohol intake. Proportional hazards models were used to estimate adjusted relative risks of hospitalization separately for 25 common types of admission.

**Results:**

During an average of 9.2 years follow-up, there were 2,834,016 incident hospital admissions. In women with BMIs (in kg/m^2^) of <22.5, 22.5 to <25, 25 to <30, 30 to <35 and 35+ standardized admission rates (and 95% confidence intervals (CIs)) per woman over a 10-year period were 2.4 (2.4 to 2.4), 2.4 (2.3 to 2.4), 2.6 (2.6 to 2.6), 3.0 (3.0 to 3.0) and 3.5 (3.4 to 3.5), respectively (*P*-value for heterogeneity <0.001). The relative increase in admission rates per 5 kg/m^2^ increase in BMI was 1.12 (1.12 to 1.13). This relationship did not vary materially by age. Corresponding average durations of stay (in days) per hospital visit within the same categories of BMI were: 3.1 (3.1 to 3.2), 2.8 (2.7 to 2.8), 2.9 (2.9 to 2.9), 3.2 (3.1 to 3.2) and 3.8 (3.7 to 3.8), respectively (*P* <0.001).

Significant increases in the risk of admission with increasing BMI were observed for 19 of the 25 types of hospital admission considered. BMI was most strongly associated with admissions with diabetes, knee-replacement, gallbladder disease and venous thromboembolism, but marked associations were found with many other common categories of admission including cataracts, carpal tunnel syndrome and diverticulitis.

**Conclusions:**

Among women 50- to 84-years old in England, around one in eight hospital admissions are likely to be attributable to overweight or obesity, translating to around 420,000 extra hospital admissions and two million extra days spent in hospital, annually.

## Background

Rates of obesity have risen substantially in most countries over the last few decades and recent estimates from the UK [1] indicate that almost one quarter of men and women are obese. The relationship between adiposity and the incidence of a number of chronic diseases and cancers, many of which commonly require hospitalization, is well documented. In particular, there is conclusive evidence for causal associations between body mass index (BMI) and type 2 diabetes [2], vascular disease [2], osteoarthritis [2], gallbladder disease [2], respiratory disease [2] and certain cancers [2,3]. However, there is still relatively little direct information on the overall impact of overweight and obesity on the use of health services.

Several studies have assessed the likely impact of projected rates of overweight and obesity on use of healthcare services worldwide [4,5] but most have been based on published estimates of attributable risk for the incidence of a relatively small number of conditions known to be strongly associated with BMI, such as type 2 diabetes, ischemic heart disease, stroke and certain cancers. One such report [4] estimated that obesity accounted for up to 2.8% of healthcare expenditure globally but concluded that this was likely to be an underestimate. Although some studies have looked directly at the relationship between BMI and hospital admission rates [6-14], relatively few have considered duration of stay [6-8,10] and most have had insufficient power to assess reliably the relationship between BMI and less common causes of hospitalization.

We aim to describe and quantify the relationship between BMI and overall rates of hospitalization, and durations of hospital stay, using routinely collected hospital admission records in a large UK cohort of middle-aged women. We also aim to assess the relationship between BMI and risk of hospitalization separately for the 25 most common categories of admission in this group of women.

## Methods

### Study population and definitions

Between 1996 and 2001, around 1.3 million women, 50- to 64-years old, were recruited into the Million Women Study through National Health Service (NHS) breast screening clinics, constituting around a quarter of all women in the UK in the eligible age range (50 to 64). Compared with the general population, study participants were slightly more likely to be current users of hormone replacement therapy (HRT) (33% versus 30%) and were slightly less likely to come from the most deprived tertile of the general population (28% versus 33%), reflecting the characteristics of women screened by the NHS Breast Screening Program. Study participants completed a questionnaire about anthropometric, social and demographic factors, and other personal characteristics, and were re-surveyed about three and eight years later to update information on various factors including weight (see [15] for further details of the study and questionnaires). Height and weight have also been measured in a sample of 3,745 women approximately eight years after recruitment.

Study participants were linked to NHS Central Registers for information on deaths, cancer registrations and emigrations and to cause-specific information on NHS hospital admission databases including inpatient and day-case admissions. For participants in England, Hospital Episodes Statistics [16] data were available from 1 April 1997; for participants in Scotland, the Scottish Morbidity Records [17] were available from 1 January 1981. Information on diagnoses and procedures associated with each hospital admission were coded to the World Health Organization’s International Classification of Diseases, 10th revision (ICD-10) [18] for diagnoses and to the Office of Population Censuses and Surveys classification of surgical operations and procedures, fourth revision (OPCS-4) [19] for procedures.

Ethics approval for the study was provided by the Eastern Multi-centre Research Ethics Committee and all participants gave signed consent for follow-up. Access to hospital admission data was approved by the Information Centre for Health and Social Care (England) and the Information Services Division (Scotland).

Admissions were classified according to whether the relevant diagnosis and/or operation codes were listed among the primary diagnosis or operation codes recorded during that hospital admission. Diabetes was not among the 25 most common primary diagnoses associated with a hospital admission, but is often an underlying reason for admission and frequently included among the other diagnoses listed for an admission. We, therefore, also considered the category of hospital admissions with any diagnosis of diabetes. Although it is not possible to separate cases of type 1 and 2 diabetes on the basis of ICD-10 code alone, since all incident admissions included in these analyses are among women at least 50 years old, it can be reasonably assumed that the vast majority of admissions for diabetes included here are due to type 2 diabetes. Some common types of admission, such as admission for hysterectomy, were not considered separately here, as they were likely to be associated with a diverse range of underlying conditions.

At recruitment, women were asked for their current weight and height and these variables were then used to derive BMI (in kg/m^2^), which was categorized as: <22.5, 22.5 to 24.9 (reference group), 25.0 to 29.9, 30 to 34.9 and 35 kg/m^2^ or more.

### Statistical analysis

Women were excluded from analyses if their BMI was unknown. In analyses of specific types of hospital admission, women were also excluded if they had a registration of cancer (other than non-melanoma skin cancer, C44) or any record of having had the relevant diagnosis or operation (whether self-reported or via hospital admission data) prior to the beginning of follow-up. Women were also excluded from analyses of admissions for uterine fibroids if they had any record of having had a hysterectomy prior to the beginning of follow-up.

In analyses of all hospital admissions, women contributed person-years from their date of recruitment (except for the 5% of women in England recruited before hospital admissions were available, for whom person-years were calculated from 1 April 1997 instead) until their date of death, emigration or the end of follow-up for hospital admissions (31 March 2008 in England and 31 December 2008 in Scotland). Each woman’s time on study was divided into intervals according to attained age, and the number of hospital admissions within each interval was treated as a Poisson variable. In order to take account of the lack of independence between successive hospital admissions within a given individual, generalized estimating equations [20] were used to generate standardized rates of hospital admissions by BMI category, and trends in rate ratios of hospital admissions per 5 unit increase in BMI, adjusted for age, region of recruitment (10 regions corresponding to the areas covered by the cancer registries), quintiles of socio-economic status based on deprivation index [21], parity and age at first birth (nulliparous, parous women cross classified by parity (1,2,3+) and age at first birth (<25, 25–29, 30+)), smoking (never, past, current), HRT use (never, past, current) and alcohol intake (never, <7 units per week, 7+ units per week). Women with missing values for any of the adjustment variables were assigned to a separate category for that variable. The main analyses were also repeated after additional adjustment for physical activity (never/rarely, once per week or less, more than once per week). Reported standard errors were adjusted for the estimated degree of over-dispersion [20]. Adjusted mean durations of stay (in days) per hospital admission were also estimated according to BMI.

In analyses with respect to specific categories of hospital admission, women contributed person-years until the date of their first relevant hospital admission, cancer registration (other than the cancer of interest or non-melanoma skin cancer), death, emigration or the end of follow-up for hospital admissions. For each of the 25 most common types of hospital admission, relative risks of admission according to BMI were estimated using proportional hazards models with attained age as the underlying time variable, stratified by region of recruitment, and adjusted for all other confounders included in analyses of all admissions described above. Analyses were repeated with categories of admission based on any operation or diagnosis rather than just those listed as the primary reason(s) for admission.

Estimates of log-linear trends in rate ratios or relative risks per 5 unit increase in BMI were corrected for measurement error in self-reported BMI, and for changes in BMI over time, using a regression dilution approach [22]. This was done by assigning to each BMI category the mean BMI value in the sample of women in which weight and height had been measured (n = 3,745) and treating these values as a continuous variable. For those categories of hospital admission where there appeared to be a J-shaped relationship with BMI, the corresponding trend statistic was estimated after exclusion of women with a BMI of less than 22.5 kg/m^2^. Analyses were repeated separately within never-HRT users and never-smokers. Since it is possible that weight change associated with some of these conditions may have occurred prior to clinical diagnosis, the main analyses were repeated after exclusion of the first three years of follow-up. Analyses of BMI in relation to all hospital admissions were also examined by attained age.

In analyses involving comparison of risks across more than two categories, variances were estimated by treating the relative risks as floating absolute risks [23].

For all hospital admissions, and for those categories of admission that were found to increase significantly with increasing BMI, the proportions of admissions in women in England 50- to 84-years old attributed to being overweight and/or obese, were estimated. We did this by applying the relative risks and rate ratios estimated here to combined national population data on BMI in women 50- to 84-years old from annual Health Surveys for England between 2006 and 2010 [1,24-27]. We also used our estimates of the average duration of stay (in days) per admission according to BMI to estimate the number of NHS bed days attributable to overweight and obesity among women in England 50- to 84-years old.

## Results

Out of a total 1,322,890 potentially eligible study participants, 71,271 were excluded because of missing information on weight and/or height. Among the 1,251,619 remaining women included in the analyses, the average age at recruitment was 56.2 years. During an average follow-up of 9.2 years, there were 2,834,016 hospital admissions. Table 1 shows characteristics of the women by category of self-reported BMI. Most women (46%) had a BMI of <25 kg/m^2^, 36% were overweight and 18% were obese. Overweight women tended to be of lower socioeconomic status and were less likely to smoke; they were also less likely to use HRT, take strenuous exercise, or drink alcohol (*P* <0.001 for heterogeneity by BMI category in each case).

**Table 1:** Characteristics of the study population at recruitment, and details of follow-up, by body mass index

	**Body mass index:**					
	**<22.5**	**22.5-24.9**	**25.0-29.9**	**30.0-34.9**	**35+**	**All women**
Number of women	241,773	337,095	447,971	159,330	65,450	1,251,619
Median (interquartile range) BMI	21.2 (1.6)	23.8 (1.3)	26.9 (2.3)	31.7 (2.3)	37.6 (4.3)	25.4 (5.6)
Mean (SD) age at recruitment	55.8 (4.9)	56.0 (4.9)	56.4 (4.9)	56.3 (4.8)	55.8 (4.6)	56.2 (4.9)
Upper third of socioeconomic group (%)	36.8	36.6	32.7	27.6	22.9	33.4
Mean (SD) number of children	2.0 (1.2)	2.1 (1.2)	2.2 (1.2)	2.3 (1.3)	2.3 (1.4)	2.1 (1.2)
Strenuous physical activity > once a week (%)	26.7	24.1	19.4	14.9	12.2	21.1
Mean (SD) alcohol intake (g/day)	6.7 (8.0)	6.6 (7.7)	5.7 (7.4)	4.6 (6.9)	3.6 (6.3)	5.9 (7.6)
Current smoker (%)	25.3	20.6	19.2	17.0	15.3	20.3
Current HRT user (%)	37.4	35.8	32.9	29.4	24.7	33.7
Hysterectomy (%)	21.1	23.5	26.6	29.0	27.2	25.0
Follow-up						
Woman-years of follow-up (1,000 s)	2,231.4	3,122.7	4,130.1	1,460.7	594.1	11,539.0
Total number incident hospital admissions	485,566	683,385	1,031,052	429,321	204,692	2,834,016
Woman-years of follow-up to first admission (1000 s)	1,484.9	2,047.6	2,577.5	852.9	327.1	7,290.0
Number women with at least one incident admission	138,740	197,724	281,238	107,813	47,160	772,675

**Figure 1:**
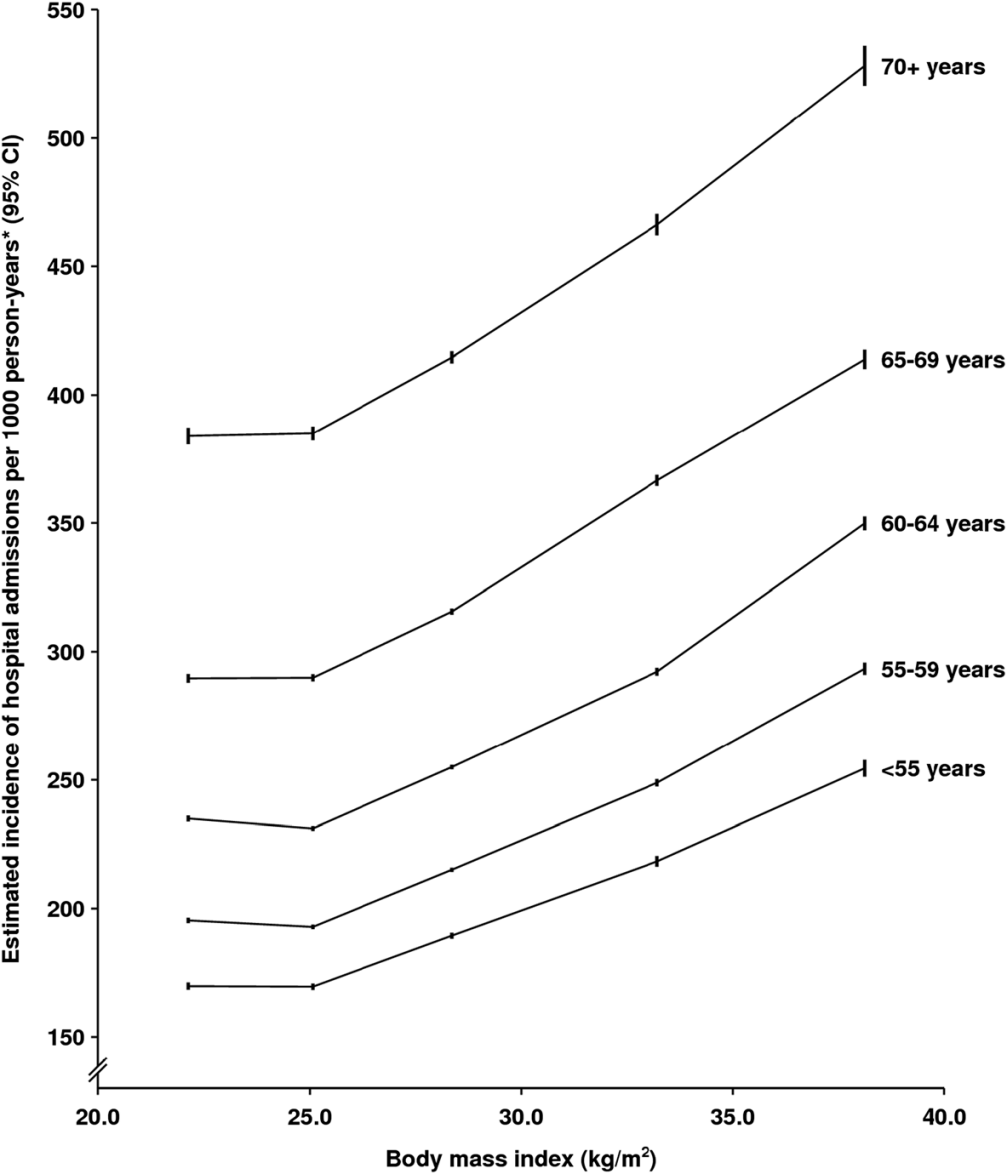
**Estimated number of hospital admissions per 1,000 person-years, by age-group and body mass index.** *Rates are standardised for age, region of residence, deprivation index, smoking, HRT use, alcohol intake, parity and age at first birth.

Among women with a BMI of 22.5 to <25 kg/m^2^, the standardized rate for all hospital admissions per 1,000 person-years was 235 (95% CI: 234 to 236): corresponding rates among women with BMIs of 25 to <30 kg/m^2^, 30 to <35 kg/m^2^, and <35 + kg/m^2^ were all significantly greater at 258 (258 to 259), 297 (296 to 298) and 346 (344 to 349), respectively (Table 2). Rates of hospitalization among women with a BMI of 30 to <35 kg/m^2^ and 35 + kg/m^2^ were also significantly greater that those among overweight women (*P* <0.001 in each case). In women with a BMI of less than 22.5 kg/m^2^ the corresponding rate was slightly higher than that in the reference group (237, 236 to 238) (Table 1). The estimated trend in the incidence rate ratio across all five BMI categories was 1.12 (1.12 to 1.13) per 5 kg/m^2^; this estimate was virtually unchanged after exclusion of the first three years of follow-up, after restriction to never-smokers and never-HRT users, and after additional adjustment for physical activity; nor was there any discernible difference in the estimated rate ratio for total admissions per 5 unit increase in BMI according to calendar period of admission (1996 to 2002, 2003 to 2008). There was significant heterogeneity by attained age in the trend in hospitalization rates per 5 kg/m^2^ (*P* <0.001) but this was largely due to a slightly smaller trend in women 75 years old or older and did not reflect substantial differences in the relationship across age-groups (Figure 1). The average adjusted durations of hospital stay (in days) per admission among women with BMIs of <22.5, 22.5 to <25, 25 to <30, 30 to <35 and 35 + kg/m^2^ were: 3.12 (3.09 to 3.15), 2.77 (2.74 to 2.79), 2.89 (2.87 to 2.91), 3.17 (3.14 to 3.20) and 3.76 (3.71 to 3.80), respectively (*P* <0.001 for heterogeneity).

**Table 2:** **Estimated standardized rates**^**a **^**for all hospital admissions, by BMI**

	**Annual standardized hospital admission rates (95% CI) per 1,000 women with body mass index (kg/m**^**2**^**):**					
Age-group	<22.5	22.5-24.9	25.0-29.9	30.0-34.9	35+	Trend in rate ratio (95% CI) per 5 kg/m^2^ increase in BMI
All women	237 (236-238)	235 (234-236)	258 (258-259)	297 (296-298)	346 (344-349)	1.12 (1.12-1.13)
50 to 54	170 (170-172)	170 (168-172)	189 (188-191)	218 (215-222)	254 (250-260)	1.13 (1.12-1.14)
55 to 59	195 (194-197)	193 (192-194)	215 (214-216)	249 (247-251)	293 (290-297)	1.13 (1.12-1.13)
60 to 64	235 (233-237)	231 (230-233)	255 (254-256)	292 (290-295)	350 (346-354)	1.13 (1.13-1.13)
65 to 69	290 (287-292)	290 (288-292)	316 (314-317)	367 (363-370)	414 (408-420)	1.13 (1.13-1.14)
75+	384 (379-389)	385 (381-389)	415 (411-418)	466 (460-473)	528 (516-540)	1.06 (1.06-1.07)

^a^Standardized for age, region of recruitment, socioeconomic status, parity, age at first birth, smoking status, hormonal therapy use and alcohol intake. BMI, body mass index; CI, confidence interval.

**Figure 2:**
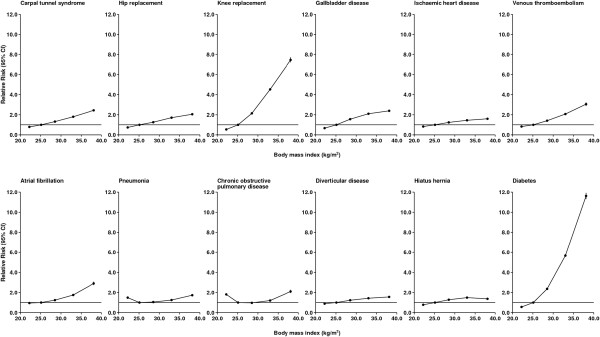
Relative risk of admission to hospital with specific diagnoses/procedures according to body mass index.

For each of 25 common categories of hospital admission, Table 3 presents the number of first hospital admissions and the relative risk (RR) of hospitalization by BMI. The most common reason for admission was cataracts with 51,750 admissions; other common reasons for admission were gallbladder disease (35,058), breast cancer (34,307) and ischemic heart disease (32,483). All but 3 of the 25 categories of admission considered showed clear associations with BMI, although the magnitudes, and sometimes the shape, of the associations varied considerably. The relationship between BMI and admission to hospital is presented graphically in Figure 2 for selected categories of hospital admission.

**Table 3:** **Relative risk**^**a **^**of first admission to hospital with 25 common categories of diagnosis/procedure, by BMI**

	**RR (95% FCI) for incident hospital admissions in women with BMI (kg/m**^**2**^**) *Number of hospital admissions***					
**Reason for admission (ICD-10 diagnosis code(s) and/or OPCS-4 procedure code(s))**	**<22.5**	**22.5 to 24.9**	**25 to 29.9**	**30.0 to 34.9**	**35+**	**Trend (95%CI)/5 kg/m**^**2**^
Circulatory disease						
Ischemic heart disease (ICD-10: I20-I25)	0.83 (0.81-0.86) *4,561*	1.0 (0.98-1.02) *7,591*	1.24 (1.22-1.26) *12,836*	1.45 (1.41-1.49) *5,262*	1.60 (1.54-1.67) *2,233*	1.25 (1.23-1.26) *P* <0.001
Atrial fibrillation (ICD-10: I48)	0.95 (0.91-1.00) *1,548*	1.0 (0.96-1.04) *2,346*	1.24 (1.20-1.28) *3,993*	1.75 (1.67-1.82) *1,963*	2.90 (2.74-3.07) *1,228*	1.43 (1.40-1.46) *P* <0.001
Stroke (ICD-10: G45, I60-I69)	1.03 (0.99-1.07) *2,984*	1.0 (0.97-1.03) *3,957*	1.13 (1.10-1.15) *6,161*	1.28 (1.23-1.33) *2,504*	1.55 (1.46-1.64) *1,189*	1.14 (1.12-1.16) *P* < 0.001
Venous thromboembolism (ICD-10: I26, I80-I82)	0.83 (0.79-0.88) *1,179*	1.0 (0.96-1.05) *1,966*	1.41(1.37-1.46) *3,754*	2.07 (1.98-2.16) *1,941*	3.05 (2.87-3.23) *1,126*	1.53 (1.49-1.56) *P* <0.001
Varicose veins (OPCS-4: L84-L88)	0.95 (0.91-0.98) *2,901*	1.0 (0.97-1.03) *4,378*	1.04 (1.02-1.07) *6,067*	0.96 (0.92-1.01) *1,997*	0.80 (0.74-0.86) *676*	0.98 (0.96-1.00) *P* = 0.03
Hemorrhoids (OPCS-4: H51-H53)	0.92 (0.88-0.96) *1,912*	1.0 (0.96-1.04) *3,001*	1.03 (1.00-1.06) *4,175*	1.03 (0.97-1.08) *1,495*	1.02 (0.94-1.11) *602*	1.04 (1.01-1.06) *P* = 0.002
Respiratory disease						
Pneumonia (ICD-10: J18)^b^	1.49 (1.43-1.55) *2,607*	1.0 (0.96-1.04) *2,383*	1.05 (1.02-1.09) *3,530*	1.25 (1.19-1.32) *1,525*	1.73 (1.62-1.85) *852*	1.22 (1.19-1.26) *P* <0.001
Chronic obstructive pulmonary disease (ICD-10: J44)^b^	1.81 (1.74-1.89) *2,292*	1.0 (0.95-1.05) *1,616*	0.97 (0.93-1.01) *2,215*	1.20 (1.12-1.28) *980*	2.10 (1.95-2.27) *678*	1.30 (1.25-1.34) *P* <0.001
Digestive disease						
Diverticular disease (ICD-10:K57)	0.89 (0.86-0.91) *3,779*	1.0 (0.98-1.03) *6,100*	1.23 (1.21-1.26) *10,313*	1.43 (1.38-1.47) *4,211*	1.56 (1.49-1.63) *1,776*	1.22 (1.20-1.24) *P* <0.001
Diaphragmatic hernia (ICD-10: K44)	0.78 (0.75-0.82) *2,635*	1.0 (0.97-1.03) *4,758*	1.28 (1.25-1.31) *8,250*	1.49 (1.44-1.54) *3,466*	1.37 (1.29-1.44) *1,294*	1.23 (1.21-1.25) *P* <0.001
Peptic ulcer (ICD-10: K25-K28)	1.06 (1.01-1.12) *1,513*	1.0 (0.96-1.05) *1,966*	1.14 (1.10-1.18) *3,115*	1.24 (1.17-1.31) *1,226*	1.49 (1.37-1.62) *602*	1.12 (1.09-1.14) *P* <0.001
Gallbladder disease (ICD-10: K80-K81; or OPCS-4: J18)	0.67 (0.65-0.70) *3,327*	1.0 (0.98-1.02) *6,950*	1.56 (1.53-1.58) *14,568*	2.10 (2.05-2.15) *7,003*	2.39 (2.30-2.47) *3210*	1.50 (1.48-1.52) *P* <0.001
Musculo-skeletal disease						
Wrist fracture (ICD-10: S52.5, S52.6, S62.0, S62.1, S62.8)	1.12 (1.08-1.17) *2,465*	1.0 (0.97-1.04) *3,162*	0.84 (0.81-0.87) *3,640*	0.72 (0.68-0.76) *1,121*	0.60 (0.54-0.66) *372*	0.81 (0.79-0.83) *P* <0.001
Hip fracture (ICD-10: S72.0, S72.1, S72.2)	1.66 (1.59-1.73) *2,177*	1.0 (0.95-1.05) *1,810*	0.79 (0.76-0.83) *2,007*	0.59 (0.54-0.65) *536*	0.60 (0.52-0.69) *210*	0.65 (0.63-0.67) *P* <0.001
Ankle fracture (ICD-10: S82.3, S82.5, S82.6, S82.8)	0.66 (0.62-0.70) *877*	1.0 (0.96-1.05) *1,869*	1.30 (1.26-1.35) *3,233*	1.62 (1.53-1.71) *1,399*	1.49 (1.37-1.63) *513*	1.32 (1.29-1.36) *P* <0.001
Hip replacement (ICD-10: M16 and OPCS-4: W37.1, W38.1, W39.1)	0.74 (0.71-0.76) *2,544*	1.0 (0.97-1.03) *4,953*	1.26 (1.24-1.29) *8,542*	1.71 (1.66-1.77) *4,006*	2.05 (1.96-2.15) *1,804*	1.39 (1.37-1.41) *P* <0.001
Knee replacement (ICD-10: M17 and OPCS-4: W40.1, W41.1, W42.1)	0.54 (0.51-0.58) *994*	1.0 (0.96-1.04) *2,690*	2.14 (2.09-2.18) *7,955*	4.52 (4.41-4.64) *5,866*	7.45 (7.21-7,71) *3,563*	2.21 (2.18-2.24) *P* <0.001
Cancers						
Non-melanoma skin cancer (ICD-10: C44)	1.10 (1.07-1.15) *3,008*	1.0 (0.97-1.03) *3,910*	0.86 (0.84-0.89) *4,575*	0.85 (0.81-0.89) *1,563*	0.77 (0.71-0.84) *541*	0.88 (0.86-0.90) *P* <0.001
Benign colon cancer (ICD-10: D12)	0.97 (0.93-1.01) *2,388*	1.0 (0.97-1.03) *3,422*	1.06 (1.03-1.09) *4,881*	1.13 (1.08-1.18) *1,831*	1.15 (1.06-1.23) *730*	1.06 (1.04-1.09) *P* <0.001
Breast cancer (ICD-10: C50)	0.93 (0.90-0.95) *6,101*	1.0 (0.98-1.02) *9,051*	1.07 (1.06-1.09) *12,644*	1.14 (1.11-1.17) *4,623*	1.18 (1.13-1.23) *1,888*	1.09 (1.07-1.10) *P* <0.001
**Other**						
Diabetes (ICD-10: E10-E11, E13-E14)^c^	0.56 (0.53-0.60) *1,109*	1.0 (0.96-1.04) *2,732*	2.36 (2.31-2.41) *8,964*	5.67 (5.54-5.80) *7,530*	11.62 (11.31-11.93) *5,720*	2.53 (2.50-2.57) *P* < 0.001
Uterine fibroids (ICD-10: D25 and OPCS-4: Q10, Q18, Q074, Q089, or Q171)	0.85 (0.81-0.89) *1,532*	1.0 (0.96-1.04) *2,360*	1.09 (1.05-1.13) *3,071*	1.18 (1.12-1.26) *1,119*	1.22 (1.12-1.34) *498*	1.13 (1.10-1.16) *P* < 0.001
Cataracts (ICD-10: H25-H26 or OPCS-4: C71-C75)	1.02 (1.00-1.04) *9,089*	1.0 (0.98-1.02) *12,713*	1.06 (1.05-1.08) *18,882*	1.23 (1.20-1.26) *7,739*	1.40 (1.35-1.45) *3,327*	1.11 (1.10-1.12) *P* < 0.001
Female genital prolapse (ICD-10: N81 and OPCS-4: M51-M53, P22-P24)	0.73 (0.71-0.76) *4,187*	1.0 (0.98-1.02) *8,480*	1.09 (1.07-1.11) *12,815*	0.99 (0.96-1.02) *4,264*	0.70 (0.66-0.74) *1,223*	1.04 (1.02-1.05) *P* < 0.001
Carpal tunnel syndrome (ICD-10: G56.0 or OPCS-4: A65.1)	0.79 (0.76-0.82) *2,874*	1.0 (0.97-1.03) *5,076*	1.32 (1.29-1.34) *8,851*	1.80 (1.75-1.86) *4,287*	2.44 (2.34-2.54) *2,346*	1.43 (1.41-1.45) *P* < 0.001

^a^Adjusted for age, geographical region, socio-economic status, age at first birth, parity, smoking status, alcohol intake, physical activity and, where appropriate, time since menopause and HRT use; ^b^trend estimated is based on women with a BMI of 22.5+ kg/m^2^; ^c^categorization based on any rather than primary diagnoses. BMI, body mass index; HRT, hormone replacement therapy; ICD-10, International Classification of Diseases, 10^th^ revision; OPCS, Office of Population Censuses and Surveys classification of surgical operations and procedures, fourth revision; RR, relative risk.

Most types of hospital admission showed a steady increase in risk across successive categories of BMI from <22.5 to 35 + kg/m^2^ (Table 3). Women with a BMI of 35 + kg/m^2^ were at least 11 times more likely to be admitted with diabetes than women in the reference group and were also at a substantially increased risk of admission with knee replacement (RR = 7.45, 7.21 to 7.71), venous thromboembolism (RR = 3.05, 2.87 to 3.23), atrial fibrillation (RR = 2.90, 2.74 to 3.07), gallbladder disease (RR = 2.39, 2.30 to 2.47), carpal tunnel syndrome (RR = 2.44, 2.34 to 2.54) and hip replacement (RR = 2.05, 1.96 to 2.15), compared with women in the reference category.

For both chronic obstructive pulmonary disease (COPD) and pneumonia, the risk of admission was significantly elevated in women with a BMI of <22.5 kg/m^2^ compared with the reference group (RR and 95% CI: 1.81, 1.74 to 1.89; and 1.49, 1.43 to 1.55, respectively) but then increased significantly across higher BMI categories. The significant excess risks observed for both conditions in women with a relatively low BMI remained after restricting analyses to never-smokers and after excluding the first three years of follow-up (data not shown).

Women with a BMI of 35 + kg/m^2^ had a significantly lower risk of admission for wrist fracture (0.60, 0.54 to 0.66), hip fracture (0.60, 0.52 to 0.69) and non-melanoma skin cancer (0.77, 0.71 to 0.84) compared with women in the reference group. The only conditions for which hospitalization was not clearly associated with BMI were hemorrhoids, varicose veins and female genital prolapse.

**Figure 3:**
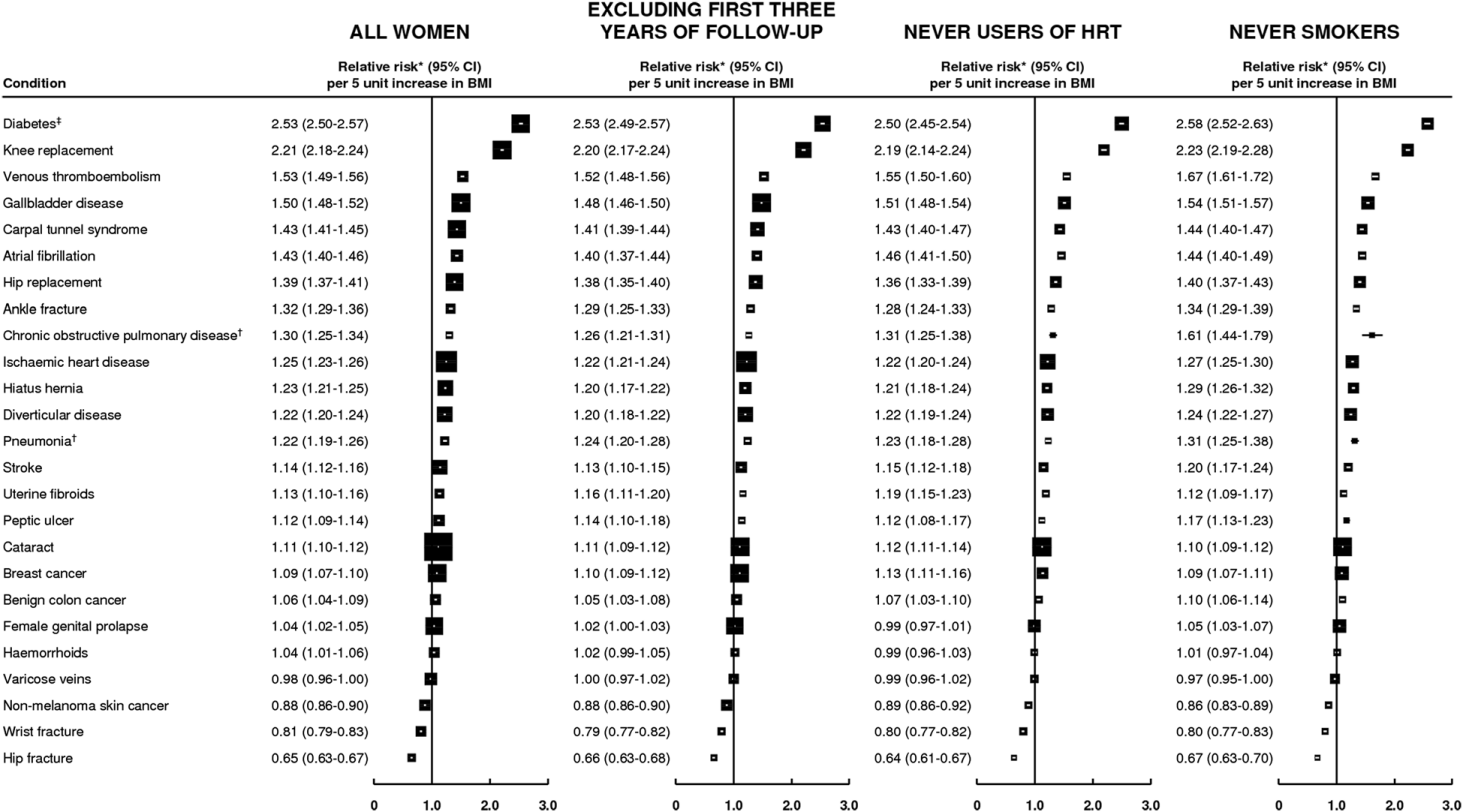
**Estimated increase in relative risk of admission to hospital with specific diagnoses/procedures per 5 unit increase in body mass index.** *Adjusted for age, geographical region, socio-economic status, age at first birth, smoking status, alcohol intake, physical activity and, where appropriate, time since menopause and HRT use. ^†^Denotes trend in women with a BMI of 22.5 kg/m^2^ or greater; ^‡^admission category based on all rather than just primary diagnoses and/or procedures.

Figure 3 presents, for each category of hospital admission, the estimated trend in relative risk per 5 kg/m^2^ increase in BMI in order of decreasing magnitude. Estimates presented for pneumonia and COPD are based on women with a BMI of 22.5 kg/m^2^ or greater. Most of these relative risks were essentially unchanged after various restrictions. There were, however, a few minor exceptions. The trend in risk of hospitalization with uterine fibroids was somewhat greater in never-HRT users than in all women (RR = 1.19 versus 1.13); corresponding trends for both venous thromboembolism and stroke were greater in never-smokers than in all women (RRs = 1.67 versus 1.53 and 1.20 versus 1.14, respectively); and corresponding trends for pneumonia and COPD (among women with a BMI of 22.5 + kg/m^2^) were greater in never-smokers than in all women (RRs = 1.31 versus 1.22 and 1.61 versus 1.30, respectively).

Relative risk estimates for specific categories of admissions based on any diagnosis or procedure associated with a hospital admission were generally very similar to those based on primary diagnoses and/or procedures alone [see Additional file 1: Webtable 1]. Estimated trends in risk per 5 kg/m^2^ for uterine fibroids, ischemic heart disease, COPD, hiatus hernia and peptic ulcer were slightly greater when based on any diagnosis or procedure but none of these increased by a factor of more than 1.07.

Estimates based on combined data from annual Health Surveys for England between 2006 and 2010 indicate that the average proportions of women in England, 50- to 84-years old, with BMIs in kg/m^2^ of less than 22.5, 22.5 to <25, 25 to <30, 30 to <35 and 35+, during that period, were around 13%, 18%, 38%, 20%, and 11%, respectively. Given these figures, we estimate that the proportion of hospital admissions in England in women 50- to 84-years old attributable to being overweight is around 3% and to being obese is around 9%. Application of these results to HES data from 2010 to 2011 [15] on total hospital admissions in England among women 50- to 84-years old suggest that around 110,000 hospital admissions per year in women of this age are due to overweight and 310,000 are due to obesity. When combined with our estimates of the average duration of stay per admission by BMI, these figures suggest that each year, approximately two million days of hospital stay in England among women 50- to 84-years old are due to overweight or obesity.

The proportion of hospital admissions attributable to overweight or obesity varies considerably by the reason for admission (Table 4) with excess weight accounting for an estimated 74% of admissions with diabetes, 66% of admissions for knee replacement and around 38% of admissions for gallbladder disease and venous thromboembolism.

**Table 4:** Estimated proportion of hospital admissions for specific diagnoses and/or operations, attributable to overweight and obesity in middle-aged UK women

**Type of admission (ICD-10 code and/or OPCS-4 code)**	**Proportion (%) of admissions attributable to BMI (kg/m**^**2**^**)**^**a**^**:**	
	**25+ (overweight/obese)**	**30+ (obese)**
Ischemic heart disease (ICD-10: I20-I25)	20	13
Atrial fibrillation (ICD-10: I48)	31	25
Stroke (ICD-10: G45, I60-I69)	14	10
Venous thromboembolism (ICD-10: I26, I80-I82)	38	28
Pneumonia (ICD-10:J18)	12	11
Chronic obstructive pulmonary disease (ICD-10: J44)	12	13
Diverticular disease (ICD-10:K57)	19	12
Diaphragmatic hernia (ICD-10:K44)	20	12
Peptic ulcer (ICD-10: K25-K28)	13	9
Gallbladder disease (ICD-10: K80-K81 or OPCS-4: J18)	38	24
Ankle fracture (ICD-10: S82.3, S82.5, S82.6, S82.8)	23	14
Hip replacement (ICD-10: M16 and OPCS-4: W37.1, W88.1, W39.1))	27	20
Knee replacement (ICD-10:M17 and OPCS-4: W40.1, W41.1,)	66	51
Benign colon cancer (ICD-10: D12)	6	4
Breast cancer (ICD-10: C50)	7	5
Diabetes (ICD-10: E10-E11, E13-E14)	74	59
Uterine fibroids (ICD-10: D25 and OPCS-4: Q10, Q18, Q074, Q089, Q171)	9	6
Cataracts (ICD-10: H25-H26 or OPCS-4: C71-C75)	10	8
Carpal tunnel syndrome (ICD-10: G56.0 or OPCS-4: A65.1)	31	23

^a^Data on prevalence of exposure among UK women are based on combined data from annual Health Surveys for England during the period 2006 to 2010 [1], during which an average of 13%, 18%, 38%, 20% and 11% of women 55- to 84-years old had a body mass index of <20, 20 to 24.9, 25 to 29.9, 30 to 34.9, and 35+ kg/m^2^, respectively. BMI, body mass index; ICD-10, International Classification of Diseases, 10^th^ revision; OPCS, Office of Population Censuses and Surveys classification of surgical operations and procedures, fourth revision.

## Discussion

Our findings, based on more than 2.8 million incident hospital admissions among 1,251,619 middle-aged women, show clearly that overall rates of hospital admission increase with increasing BMI. Over a 10-year period, the estimated number of hospital admissions per woman among women with BMIs (in kg/m^2^) of <22.5, 22.5 to <25, 25 to <30, 30 to <35 and 35+ were 2.4, 2.4, 2.6, 3.0 and 3.5, respectively. Total admission rates were significantly higher in both overweight and obese women compared to women with a BMI of 22.5 to <25 kg/m^2^. A positive association between BMI and the risk of hospitalization was found for 19 of the 25 most common types of hospital admission within this age-group.

Few studies have examined directly the impact of overweight or obesity on overall hospitalization rates [6-14]. The largest of these, involving Australian adults 45-years old and older [14], reported overall admission rates (per 10 person-years) of 1.83 in women with a BMI of 35 to 50 kg/m^2^ compared with 1.02 in women with a BMI of 18.5 to 24.9 kg/m^2^. Two relatively small UK studies of Scottish residents [6,7] have also demonstrated significant associations between obesity and rates of acute admissions [6] and durations of hospital stays [7], but neither study reported estimates of absolute numbers of admissions due to excess weight or considered the effect of adiposity on specific types of admission. Thus, while the published evidence is consistent with ours in demonstrating a positive association between BMI and hospital admissions, the magnitudes of the reported relative risks vary somewhat across studies, perhaps reflecting differing age-ranges, populations and types of hospital admissions considered.

Many of the relationships between BMI and specific conditions described here are well established [2,3], most notably those with type 2 diabetes, ischemic heart disease, stroke, joint replacements, gallbladder disease and certain cancers. However, more modest associations found here between BMI and other common conditions are also of public health importance since many of these conditions account for substantial numbers of hospital admissions in this age-group. When we examined the total number of first admissions with any of the 19 conditions found to be positively associated with BMI in this cohort that were due to overweight or obesity, only 62% were for diabetes, ischemic heart disease, stroke, joint replacements, gallbladder disease or cancer; the remainder were largely due to admissions with venous thromboembolism, diverticular disease, diaphragmatic hernia, cataracts or carpal tunnel syndrome. Thus, published reports on the relationship between obesity and use of healthcare resources based only on those conditions most strongly related to BMI are likely to underestimate its full impact.

For those conditions whose relationship with BMI has been less well studied, our findings constitute important new etiologic evidence. For example, we are aware of only one previous study that has reported on BMI and diverticular disease in women [28] which reported significantly increased risks in overweight (RR = 1.29) and obese women (RR = 1.33), consistent with corresponding estimates from our study of 1.23 and 1.43, respectively. Another study [14] reported relative risks in men and women combined of 1.42 and 2.18 associated with overweight and obesity, respectively. There is also limited evidence with which to compare our findings for other digestive diseases, such as peptic ulcer and diaphragmatic hernia, although one study reported a significant increase in the risk of peptic ulcer in overweight and obese adults [29], and a meta-analysis of data from four studies of diaphragmatic hernia demonstrated a significantly increased risk in individuals with a BMI above the normal range [30].

Previous studies of BMI in relation to uterine fibroids have yielded conflicting results with most [31], but not all [32], reporting positive associations. We found a modest increase in risk with increasing BMI (trend in RR per 5 kg/m^2^ increase in BMI = 1.13), which was higher in never-users of HRT (RR = 1.19), consistent with the hypothesis that BMI increases the risk of uterine fibroids by increasing circulating hormone levels.

The risk of developing cataracts has been found to increase with BMI [33,34], although the extent to which this is due to subclinical and clinical diabetes is unclear [34]. In our data, the trend in risk of hospital admission with cataracts per 5 kg/m^2^ increase in BMI (1.11, 1.10 to 1.12) was somewhat attenuated after exclusion of women with previous diabetes (RR = 1.06, 1.05 to 1.07) suggesting that the effect of BMI on cataract risk may be, at least partly, driven by its effect on diabetes. Several previous studies have found a positive association between BMI and carpal tunnel syndrome [35-37]. The largest of these [37], involving 3,391 cases of carpal tunnel syndrome, reported a two-fold increase in risk among obese individuals 50- to 79-years old, similar to our estimated relative risk of 2.44 in women with a BMI of 35+ kg/m^2^. There are few studies of non-melanoma skin cancer in relation to BMI, but at least one has reported a significant reduction in the risk of basal cell cancer in those with an increased BMI [38].

Studies of the impact of overweight and obesity on the use of healthcare services vary considerably in terms of the methods used [4-14] and those aimed at assessing the economic impact have tended to consider only those health conditions widely recognized as being strongly associated with adiposity [4,5]. To our knowledge, ours is the first UK study to present estimates of the absolute numbers of hospital admissions, and days of hospital stay, attributable to excess weight. Our findings suggest that annually, around 420,000 hospital admissions and two million days of hospital stay are due to overweight or obesity among women in England 50- to 84-years old.

The major strength of this prospective study is its power to examine reliably the role of BMI not only with respect to total hospital admissions and duration of hospital stay, but also in relation to admission with specific diagnoses and/or operations that are common among middle-aged women. An obvious limitation is that the study is restricted to women, since the effects of BMI on some conditions may well differ in men. In addition, our analyses did not distinguish between elective and emergency admissions, and BMI may have a different impact on the rates of these two categories of admission. Relatively few women in our cohort reported a BMI of below 18.5 kg/m^2^ at recruitment and so, given the considerable potential for reverse causality among such women, it was not feasible to estimate reliably hospitalization risks among underweight women. The availability of information on a wide range of potential confounders, and a relatively long follow-up period (9.2 years on average), strengthen the reliability of the findings. Although our analyses are predominantly based on self-reported BMI, estimates of trends in rates and risks of hospital admissions in these analyses were calculated using measured BMI collected some eight years after recruitment in a subset of women, thus minimizing any potential biases due to measurement error or substantial changes in BMI over time. It is possible that other indices of adiposity, such as waist circumference, may show a different relationship with the risk of hospitalization, but these were not considered here. While estimates of absolute numbers of admissions and days of hospital stay attributable to overweight and obesity presented here are specific to the UK, the general patterns of association observed between BMI and the relative risks of hospitalization with various outcomes should be broadly generalizable.

## Conclusions

Around one in eight of all hospital admissions and two million days of hospital stay annually are due to overweight or obesity among women 50- to 84-years old in England. Clearly, the contribution of overweight and obesity to hospital stays across the entire UK population is likely to be considerably larger. These findings demonstrate that current levels of overweight and obesity among women in the UK are making a substantial contribution to the burden of disease and hospitalization.

## Competing interests

The authors declare that they have no competing interests.

## Authors’ contributions

GR, VB and JG conceived of the study. GR, BC and AB were responsible for study design and statistical analyses. GR drafted the manuscript. All authors read and approved the final manuscript.

## Supplementary Material

Additional file 1 Supplementary material. Click here for file
