# Supplementary material for: Hospital admissions in relation to body mass index in UK women: a prospective cohort study
Source: BMC Med. 2014 Mar 15;12:45. doi: 10.1186/1741-7015-12-45 (PMC4003825; doi:10.1186/1741-7015-12-45)
Supplement: Additional file 1 — Supplementary material. [file 1741-7015-12-45-S1.doc]

**Hospital admissions in relation to body mass index in UK women: a prospective cohort study**

**Reeves GK, Balkwill A, Cairns BJ, Green J, Beral V, for the Million Women Study Collaborators**

**Supplementary Material**

Correspondence to: Dr Gillian Reeves

Cancer Epidemiology Unit

Richard Doll Building

Oxford OX3 7LF, UK

Tel: +44-(0)-1865-289-600

e-mail: gill.reeves@ceu.ox.ac.uk

**Webtable 1. Relative risk of first admission to hospital with 25 common categories of diagnosis/procedure by BMI, based on any associated diagnoses/procedures instead of just primary diagnoses/procedures**

|  | **RR (95% FCI) for incident hospital admissions in women with BMI (kg/m^2^)**  ***No. of hospital admissions*** | | | | | |
| --- | --- | --- | --- | --- | --- | --- |
| **Reason for admission (ICD-10 diagnosis code(s) and/or OPCS-4 procedure code(s) )** | **<22.5** | **22.5-24.9** | **25-29.9** | **30.0-34.9** | **35+** | **Trend (95%CI) / 5kg/m^2^** |
| **Circulatory disease** |  |  |  |  |  |  |
| Ischaemic heart disease (ICD-10: I20-I25) | 0.86 (0.84-0.88) *6,569* | 1.0 (0.98-1.02) *10,595* | 1.25 (1.24-1.27) *18,337* | 1.58 (1.54-1.61)  *8,138* | 1.93 (1.87-1.99)  *3,839* | 1.30 (1.29-1.32) p<0.001 |
| Atrial fibrillation (ICD-10: I48) | 1.01 (0.98-1.04) *3,802* | 1.0 (0.97-1.03)  *5,373* | 1.23 (1.20-1.25)  *9,219* | 1.74 (1.69-1.79)  *4,641* | 2.74 (2.77-2.98)  *2,914* | 1.41 (1.39-1.43) p<0.001 |
| Stroke (ICD-10: G45, I60-I69) | 1.05 (1.02-1.09) *3,939* | 1.0 (0.97-1.03)  *5,123* | 1.12 (1.09-1.14) *7,986* | 1.28 (1.24-1.32)  *3,294* | 1.58 (1.50-1.66)  *1,608* | 1.14 (1.12-1.16) p<0.001 |
| Venous thromboembolism (ICD-10: I26, I80-I82) | 0.86 (0.82-0.90) *1,731* | 1.0 (0.96-1.04)  *2,804* | 1.38(1.34-1.42)  *5,250* | 1.98 (1.90-2.05)  *2,662* | 2.92 (2.78-3.07)  *1,544* | 1.49 (1.47-1.52) p<0.001 |
| Varicose veins (OPCS-4: L84-L88) | 0.95 (0.91-0.98) *2,901* | 1.0 (0.97-1.03)  *4,378* | 1.04 (1.02-1.07) *6,067* | 0.96 (0.92-1.01)  *1,997* | 0.80 (0.74-0.86)  *676* | 0.98 (0.96-1.00) p=0.03 |
| Haemorrhoids (OPCS-4: H51-H53) | 0.92 (0.88-0.96) *1,912* | 1.0 (0.96-1.04)  *3,001* | 1.03 (1.00-1.06)  *4,175* | 1.03 (0.97-1.08)  *1,495* | 1.02 (0.94-1.11)  *602* | 1.04 (1.01-1.06) p=0.002 |
| **Respiratory disease** |  |  |  |  |  |  |
| Pneumonia (ICD-10: J18)^+^ | 1.40 (1.35-1.45) *3,753* | 1.0 (0.97-1.03)  *3,641* | 1.06 (1.03-1.09)  *5,398* | 1.27 (1.22-1.32)  *2,352* | 1.78 (1.69-1.88)  *1,327* | 1.23 (1.21-1.26) p<0.001 |
| Chronic obstructive pulmonary disease (ICD-10: J44) ^+^ | 1.51 (1.47-1.56) *5,308* | 1.0 (0.97-1.03)  *4,522* | 1.07 (1.05-1.10) *6,878* | 1.46 (1.42-1.52)  *3,362* | 2.37 (2.27-2.48)  *2,111* | 1.39 (1.36-1.41) p<0.001 |
| **Digestive disease** |  |  |  |  |  |  |
| Diverticular disease (ICD-10: K57) | 0.89 (0.87-0.91) *6,504* | 1.0 (0.98-1.02) *10,481* | 1.22 (1.20-1.24) *17,636* | 1.47 (1.43-1.50)  *7,454* | 1.64 (1.58-1.70)  *3,194* | 1.24 (1.22-1.25) p<0.001 |
| Diaphragmatic hernia (ICD-10: K44) | 0.77 (0.75-0.78) *6,531* | 1.0 (0.98-1.02) *12,111* | 1.38 (1.36-1.40) *22,798* | 1.69 (1.66-1.73)  *10,079* | 1.68 (1.63-1.74)  *4,044* | 1.32 (1.31-1.33) p<0.001 |
| Peptic ulcer (ICD-10: K25-K28) | 1.04 (1.00-1.08) *2,397* | 1.0 (0.97-1.04)  *3,209* | 1.16 (1.13-1.19)  *5,172* | 1.34 (1.29-1.40)  *2,192* | 1.66 (1.57-1.77)  *1,108* | 1.17 (1.15-1.19) p<0.001 |
| Gallbladder disease (ICD-10: K80-K81; or OPCS-4: J18) | 0.70 (0.68-0.72) *4,059* | 1.0 (0.98-1.02)  *8,144* | 1.53 (1.51-1.56) *16,914* | 2.09 (2.04-2.13)  *8,256* | 2.47 (2.39-2.55)  *3,935* | 1.50 (1.48-1.51) p<0.001 |
| **Musculo-skeletal disease** |  |  |  |  |  |  |
| Wrist fracture (ICD-10: S52.5, S52.6, S62.0, S62.1, S62.8) | 1.14 (1.10-1.19)  *2,689* | 1.0 (0.97-1.03)  *3,389* | 0.85 (0.82-0.87)  *3,941* | 0.72 (0.68-0.76)  *1,202* | 0.62 (0.56-0.68)  *412* | 0.81 (0.79-0.83) p<0.001 |
| Hip fracture (ICD-10: S72.0, S72.1, S72.2) | 1.66 (1.59-1.73) *2,247* | 1.0 (0.96-1.05)  *1,864* | 0.79 (0.76-0.83)  *2,071* | 0.60 (0.56-0.66)  *561* | 0.62 (0.54-0.71)  *224* | 0.65 (0.63-0.68) p<0.001 |
| Ankle fracture (ICD-10: S82.3, S82.5, S82.6, S82.8) | 0.67 (0.63-0.71) *940* | 1.0 (0.96-1.05) *1,969* | 1.30 (1.26-1.35) *3,405* | 1.61 (1.53-1.70) *1,472* | 1.49 (1.37-1.62) *540* | 1.32 (1.28-1.35) p<0.001 |
| Hip replacement (ICD-10: M16 and OPCS-4: W37.1, W38.1, W39.1) | 0.74 (0.71-0.77) *2,584* | 1.0 (0.97-1.03)  *4,993* | 1.26 (1.24-1.29)  *8,615* | 1.71 (1.65-1.76)  *4,028* | 2.05 (1.96-2.15)  *1,814* | 1.38 (1.36-1.41) p<0.001 |
| Knee replacement (ICD-10: M17 and OPCS-4: W40.1,  W41.1, W42.1) | 0.55 (0.51-0.58) *1,013* | 1.0 (0.96-1.04)  *2,717* | 2.13 (2.08-2.18)  *7,999* | 4.50 (4.39-4.62)  *5,893* | 7.41 (7.17-7.66)  *3,580* | 2.20 (2.17-2.24) p<0.001 |
| **Cancers** |  |  |  |  |  |  |
| Non-melanoma skin cancer (ICD-10: C44) | 1.10 (1.06-1.14) *3,050* | 1.0 (0.97-1.03)  *3,985* | 0.86 (0.84-0.89)  *4,657* | 0.84 (0.80-0.89)  *1,585* | 0.77 (0.71-0.84)  *555* | 0.88 (0.86-0.90) p<0.001 |
| Benign colon cancer (ICD-10: D12) | 0.97 (0.93-1.00) *2,938* | 1.0 (0.97-1.03) *4,229* | 1.07 (1.04-1.09) *6,096* | 1.16 (1.11-1.21) *2,382* | 1.20 (1.13-1.28) *950* | 1.08 (1.06-1.10) p<0.001 |
| Breast cancer (ICD-10: C50) | 0.93 (0.90-0.95) *6,247* | 1.00 (0.98-1.02) *9,254* | 1.08 (1.06-1.09) *12,949* | 1.15 (1.11-1.18)  *4,760* | 1.19 (1.14-1.25)  *1,954* | 1.09 (1.08-1.10) p<0.001 |
| **Other** |  |  |  |  |  |  |
| Diabetes (ICD-10: E10-E11, E13-E14) | 0.56 (0.53-0.60) *1,109* | 1.0 (0.96-1.04)  *2,732* | 2.36 (2.31-2.41)  *8,964* | 5.67 (5.54-5.80)  *7,530* | 11.62 (11.31-11.93) *5,720* | 2.53 (2.50-2.57) p<0.001 |
| Uterine fibroids (ICD-10: D25 and OPCS-4: Q10, Q18, Q074, Q089, or Q171) | 0.83 (0.80-0.86) *2,803* | 1.0 (0.97-1.03)  *4,484* | 1.11 (1.08-1.14) *6,054* | 1.24 (1.19-1.30)  *2,281* | 1.39 (1.31-1.47)  *1,081* | 1.17 (1.15-1.20) p<0.001 |
| Cataracts (ICD-10: H25-H26 or OPCS-4: C71-C75) | 1.02 (1.00-1.04)  *9,271* | 1.0 (0.98-1.02) *12,959* | 1.06 (1.05-1.08) *19,204* | 1.23 (1.21-1.26)  *7,906* | 1.40 (1.36-1.45)  *3,402* | 1.11 (1.10-1.12) p<0.001 |
| Female genital prolapse (ICD-10: N81 and OPCS-4: M51-M53, P22-P24) | 0.73 (0.71-0.75) *4,380* | 1.0 (0.98-1.02)  *8,884* | 1.10 (1.08-1.12) *13,472* | 1.01 (0.98-1.04)  *4,524* | 0.71 (0.68-0.75)  *1,297* | 1.04 (1.03-1.06) p<0.001 |
| Carpal tunnel syndrome (ICD-10: G56.0 or OPCS-4: A65.1) | 0.79 (0.76-0.82) *2,926* | 1.0 (0.97-1.03)  *5,171* | 1.31 (1.29-1.34)  *9,002* | 1.80 (1.75-1.85)  *4,370* | 2.44 (2.34-2.54)  *2,392* | 1.43 (1.41-1.45) p<0.001 |

* adjusted for age, geographical region, socio-economic status, age at first birth, parity, smoking status, alcohol intake, physical activity and, where appropriate, time since menopause and HRT use; ^+^ trend estimated is based on women with a BMI of 22.5+ kg/m^2^
